# Risk factors of early postoperative bowel obstruction for patients undergoing selective colorectal surgeries

**DOI:** 10.1186/s12876-021-02025-8

**Published:** 2021-12-18

**Authors:** Shuguang Yang, Huiying Zhao, Jianhui Yang, Youzhong An, Hua Zhang, Yudi Bao, Zhidong Gao, Yingjiang Ye

**Affiliations:** 1 Department of Critical Care Medicine, Peking University People’s Hospital, 11 Xizhimen South Street, Beijing, 100044 People’s Republic of China; 2 Research Center of Clinical Epidemiology, Peking University Third Hospital, Haidian District, Xue Yuan Road, Beijing, 100191 People’s Republic of China; 3 Laboratory of Surgical Oncology, Department of Gastrointestinal Surgery, Peking University People’s Hospital, 11 Xizhimen South Street, Beijing, 100044 People’s Republic of China

**Keywords:** Risk-factors, Early postoperative bowel obstruction, Colorectal surgery

## Abstract

**Objective:**

Postoperative bowel obstruction was one of the most severe complications in patients who received colorectal surgeries. This study aimed to explore risk factors of early postoperative obstruction and to construct a nomogram to predict the possibility of occurrence.

**Methods:**

The records of 1437 patients who underwent elective colorectal surgery in Peking University People’s Hospital from 2015 to 2020 were retrospectively collected. Risk factors of early postoperative bowel obstruction were identified by logistic regression analysis and a nomogram was then constructed. Bootstrap was applied to verify the stability of the model.

**Results:**

COPD, hypothyroidism, probiotic indications, duration of antibiotics, and time to postoperative feeding were identified as independent risk factors and were put into a nomogram for predicting early postoperative bowel obstruction. The nomogram showed robust discrimination, with the area under the receiver operating characteristic curve was 0.894 and was well-calibrated.

**Conclusion:**

A nomogram including independent risk factors of COPD, hypothyroidism, probiotic indications, duration of antibiotics, and time to postoperative feeding were established to predict the risk of early postoperative bowel obstruction.

## Introduction

Postoperative bowel obstruction, either mechanical or functional, was a common and severe complication that might affect the recovery of bowel function and lead to prolonged hospitalization and rising medical costs. Previous studies showed that postoperative bowel obstruction could even increase mortality and morbidity in patients following abdominal surgery [1, 2]. Postoperative bowel obstruction might be classified based on the time duration from the initial surgery. Early postoperative bowel obstruction was defined as occurring within the first 30 days after surgery [3]. Edna et al. found that the incidence of postoperative small bowel obstruction after the colorectal was 9% [4]. The reason causing postoperative bowel obstruction was either mechanical obstruction from adhesions or paralytic ileus in abdominal surgery [5].

Previous studies found that cystotomy, lysis of adhesions, concomitant bowel surgery postoperative transfusion were independent risk factors for the development of small bowel obstruction after benign gynecologic surgery [6]. Several studies focused on the incidence and management of postoperative bowel obstruction. The risk factors of postoperative bowel obstruction after colorectal surgery have not been adequately documented. We aimed to develop and validate a nomogram for predicting the risk factors of early postoperative obstruction for patients after colorectal surgery, which may help us identified these patients with a high risk of early postoperative obstruction and may improve the patients’ quality of life and reduce the length of hospital stay and costs of hospitalization.

## Materials and methods

This was a retrospective nested case–control study that retrospectively enrolled patients who underwent colonic and/or rectal surgery from January 2015 to December 2020 at Peking University People’s Hospital. Patients who were diagnosed with digestive tract perforation before surgery, patients with a history of gastrointestinal surgery, and patients who underwent enterostomy, as jejunostomy or ileostomy were excluded. The reason why patients who had a history of gastrointestinal surgery and a stoma with an ectopic or ileostomy were excluded was that many studies [7, 8] showed us that these patients with reconstruction of gastrointestinal may affect bowel function and we explored the risk factors of bowel function for patients underwent colorectal surgery in this study. Patients were divided into two groups according to whether clinical and radiological signs of bowel obstruction were identified or not.

### Data collection

Patients’ demographic characteristics (age, sex, Body mass index [BMI]), history of smocking or alcohol, underlying disease (hypertension, coronary heart disease [CHD], arrhythmia, cerebral infraction, encehalorrhagia, diabetes mellitus, chronic obstructive pulmonary disease [COPD], and blood disease that was diagnosed by a regular hospital, hypothyroidism [the level of thyroxine was higher than normal before this surgery], renal inadequacy [the level of creatinine was higher than normal before this surgery], hyperlipidemia [the level of triglyceride was higher than normal before this surgery], hepatic inadequacy [the Child–Pugh score was more than 6 before this surgery], preoperative chemotherapy [chemotherapy was administered within a year before this surgery], preoperative anemia [the level of hemoglobin was lower than normal before this surgery], preoperative ileus [diagnosed by abdominal imaging examination and/or manifestations include nausea and vomiting, cramps, bloating and retention of stool and flatus within 3 month before this surgery]), surgical details including American Society of Anesthesiologists [ASA] classification, bowel preparation, surgical site, surgical approach, excision method, operative time, antibiotic correlation (preoperative antibiotics, preoperative fortified antibiotics and postoperative fortified antibiotics), time to postoperative feeding, probiotic correlation (using probiotics and probiotic indications [the indications of probiotic were abdominal distension, diarrhea, constipation and indigestion]), postoperative albumin level, postoperative analgesia (no analgesia, opioids, opioids combine non-steroidal anti-inflammatory drugs [NSAIDs], and NSAIDs), time to extraction of gastric and drainage tube were recorded.

Early postoperative bowel obstruction was defined as the clinical presentation with abdominal distension or pain within 30 days after surgery [3]. The clinical diagnosis must be confirmed by the presence of dilated bowel loops with multiple fluid levels in the plain abdominal X-ray or computed tomography [CT] [9].

### Statistical analysis

SPSS 26.0 (IBM) was used for all statistical analyses. Data were given as means with standard deviations or as medians with interquartile ranges (IQRs), as appropriate. The normality of data distributions was assessed, after which non-normally distributed data were compared via Mann–Whitney U tests and normally distributed data were compared via student’s t-tests, while categorical data were compared via χ^2^ tests or Fisher’s exact test. Univariate logistic regression analyses were performed based upon whether or not bowel obstruction. Variables that attained a *p* < 0.05 in this univariate model were included in a subsequent multivariate model to identify independent predictors of bowel obstruction. For this multivariate analysis, backward stepwise elimination was conducted such that only variables yielding a *p* < 0.05 were retained for the final model. Odds ratios (OR) and corresponding 95% confidence intervals (CI) were calculated for all variables and associated relationships. For all analyses, a *p* value of less than 0.05 was considered to be statistically different.

Logistic regression was used to screen the associated factors and establish the prediction model using a stepwise method, and a nomogram was applied for convenience of clinical application. The performance of the nomogram was assessed by discrimination and calibration. The discrimination ability of the model was quantified using the area under the receiver operating characteristic curve (AUC). Bootstrap was applied to verify the stability of the model, and 1000 times of retractable extraction was adopted. The above analysis was completed by the R software RMS package.

## Results

### Patient characteristics

A total of 1437 patients (mean age, 64.24 [range 19–93] years; 47.6% female) were enrolled. The incidence of early postoperative bowel obstruction was 10.6%. The clinical characteristics of patients were shown in Table 1. There were no statistically significant differences in BMI, preoperative anemia, preoperative ileus, bowel preparation, location of colorectal resection, operative time, hypoproteinemia, postoperative analgesia, duration of postoperative analgesia, or time to the extraction of a gastric tube for patients who underwent colorectal surgery.

**Table 1:** Patient characteristics and univariable analysis of the risk of postoperative bowel obstruction

Variables	No postoperative ileus	Postoperative ileus	*χ*^2^/*t*	*P* value
N (%)	n = 1284	n = 153		
Female	615 (47.90)	69 (45.10)	0.429	0.512
Age	63.92 ± 13.16	66.97 ± 12.02	0.022	0.004
BMI	23.64 ± 3.55	23.09 ± 3.24	0.603	0.051
Right colectomy	436 (33.96)	47 (30.72)	0.642	0.423
Transverse colectomy	60 (4.67)	11 (7.19)	1.844	0.175
Left colectomy	124 (9.66)	18 (11.76)	0.682	0.409
Sigmoid colectomy	450 (35.05)	54 (35.29)	0.004	0.952
Rcctectomy	289 (22.51)	34 (22.22)	0.006	0.936
Malignant	1224 (95.33)	148 (96.73)	0.625	0.429
Hypertension	798 (62.15)	91 (59.48)	0.414	0.520
CHD	168 (13.08)	24 (15.69)	0.800	0.371
Arrhythmia	85 (6.62)	11 (7.19)	0.071	0.790
Cerebral infarction	106 (8.26)	14 (9.15)	0.143	0.705
Encepalorrhagia	8 (0.62)	2 (1.31)	0.201	0.654
Hypothyroidism	23 (1.79)	8 (5.23)	6.111	0.013
Diabetes mellitus	234 (18.22)	31 (20.26)	0.377	0.539
COPD	50 (3.89)	15 (9.80)	11.056	0.001
Renal inadequacy	33 (2.57)	4 (2.61)	0.000	1.000
Hyperlipidemia	258 (20.09)	37 (24.18)	1.401	0.236
Hepatic inadequacy	17 (1.32)	3 (1.96)	0.073	0.787
Blood disease	12 (0.93)	1 (0.65)	0.000	1.000
History of alcohol	169 (13.16)	24 (15.69)	0.749	0.387
History of smocking	218 (16.98)	28 (18.30)	0.169	0.681
Preoperative chemotherapy	52 (4.05)	6 (3.92)	0.006	0.939
Preoperative anemia	565 (44.00)	73 (47.71)	0.762	0.383
Preoperative ileus	249 (19.39)	33 (21.57)	0.421	0.810
ASA I	145 (11.29)	18 (11.76)	7.556	0.047*
ASA II	937 (72.98)	98 (64.05)		
ASA III	192 (14.95)	35 (22.88)		
ASA IV	10 (0.78)	2 (1.31)		
Soapsuds enema for bowel preparation	133 (10.36)	18 (11.76)	0.288	0.592
Oral laxatives for bowel preparation	1178 (91.74)	135 (88.24)	2.135	0.144
Glycerin enema for bowel preparation	212 (16.51)	22 (14.38)	0.456	0.500
Oral antibiotics for bowel preparation	58 (4.51)	4 (2.61)	1.199	0.274
Laparotomy	644 (50.16)	57 (37.25)	9.107	0.003
Curative resection	1126 (87.69)	132 (86.27)	0.253	0.615
Operative time (Min)	208.17 ± 67.78	205.74 ± 68.67	0.133	0.678
Preoperative antibiotics	80 (6.23)	17 (11.11)	5.173	0.023
Preoperative fortified antibiotics	45 (3.50)	12 (7.84)	6.755	0.009
Postoperative fortified antibiotics	536 (41.74)	76 (49.67)	3.515	0.061
Duration of antibiotics (day)	6.68 ± 3.62	11.92 ± 9.10	213.504	0.000
Time to postoperative feeding (day)	5.48 ± 1.75	8.07 ± 4.46	222.496	0.000
Using probiotics	348 (27.10)	54 (35.29)	4.553	0.033
Probiotic indications	505 (39.33)	150 (98.04)	189.965	0.000
Postoperative analgesia	1284	153	0.149	0.930*
None	71 (5.53)	7 (4.58)		
Opioids	1131 (88.08)	137 (8.95)		
Opioids and NSAIDs^1^	69 (5.37)	8 (5.23)		
NSAIDs	13 (1.01)	1 (0.65)		
Duration of analgesia	2.77 ± 0.98	2.99 ± 1.49	0.040	0.074
Hypoproteinemia	1067 (89.10)	134 (87.58)	2.001	0.157
Extraction of gastric tube (day)	1.74 ± 1.48	3.04 ± 9.12	86.873	0.085
Extraction of drainage tube (day)	8.77 ± 7.11	11.41 ± 5.24	5.618	0.000

BMI: body-mass index, CHD: coronary heart disease, COPD: chronic obstructive pulmonary disease, ASA: American Society of Anesthesiologists, NSAIDs: non-steroidal anti-inflammatory drugs

*Comparison between two groups, *group and*group…, the difference between groups was statistically significant (*P* < 0.01 or *P* < 0.05)

### Selected factors for model

We found that age, hypothyroidism (*p* = 0.013), COPD (*p* = 0.001), ASA classification (*p* = 0.047), laparotomy (*p* = 0.003), preoperative antibiotics (*p* = 0.023), preoperative fortified antibiotics (*p* = 0.009), duration of antibiotics (*p* < 0.001), time of first postoperative feeding (*p* < 0.001), using probiotics (*p* = 0.033), probiotic indications (*p* < 0.001) and time to extraction of drainage (*p* < 0.001) were significant difference for patients suffering early postoperative obstruction after colorectal surgery, comparing with patients did not suffering early postoperative obstruction. COPD (*p* = 0.046), hypothyroidism (*p* = 0.003), probiotic indications (*p* < 0.001), duration of antibiotics (*p* < 0.001), and time of first postoperative feeding (*p* < 0.001) were found as independent risk factors of postoperative bowel obstruction after adjusting logistic regression (Table 2).

**Table 2:** Risk factors of postoperative bowel obstruction in logistic regress

	OR (95%CI)	*P* value
COPD		0.046
No	Ref	
Yes	2.56 (1.02–1.05)	
Hypothyroidism		0.003
No	Ref	
Yes	4.92 (1.71–14.19)	
Probiotic indications		< 0.001
No	Ref	
Yes	30.26 (9.37–97.70)	
Duration of antibiotics (day)	1.10 (1.05–1.15)	< 0.001
Time to postoperative feeding (day)	1.27 (1.16–1.39)	< 0.001

### Predict model for early postoperative bowel obstruction

**Fig. 1:**
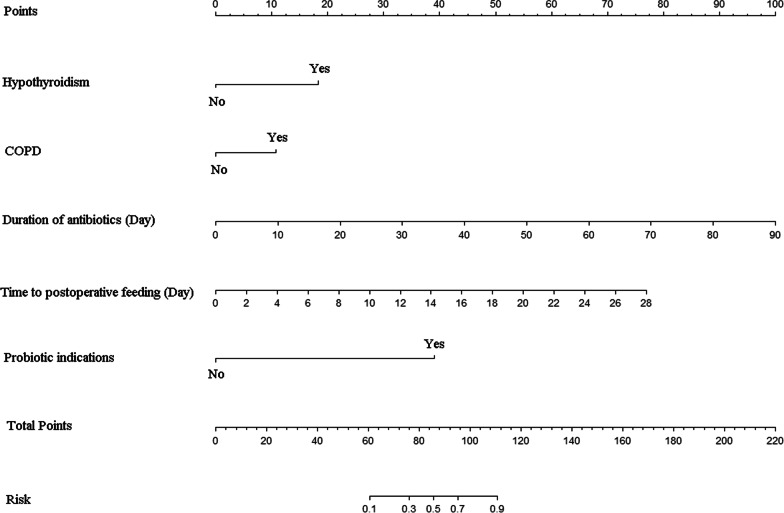
Nomogram for early postoperative bowel obstruction after colorectal surgery. The value of variable was given a score on the point scale axis. To estimate the probability of early postoperative bowel obstruction, a total score could be calculated by adding each and could be projected to the lower total point scale

**Fig. 2:**
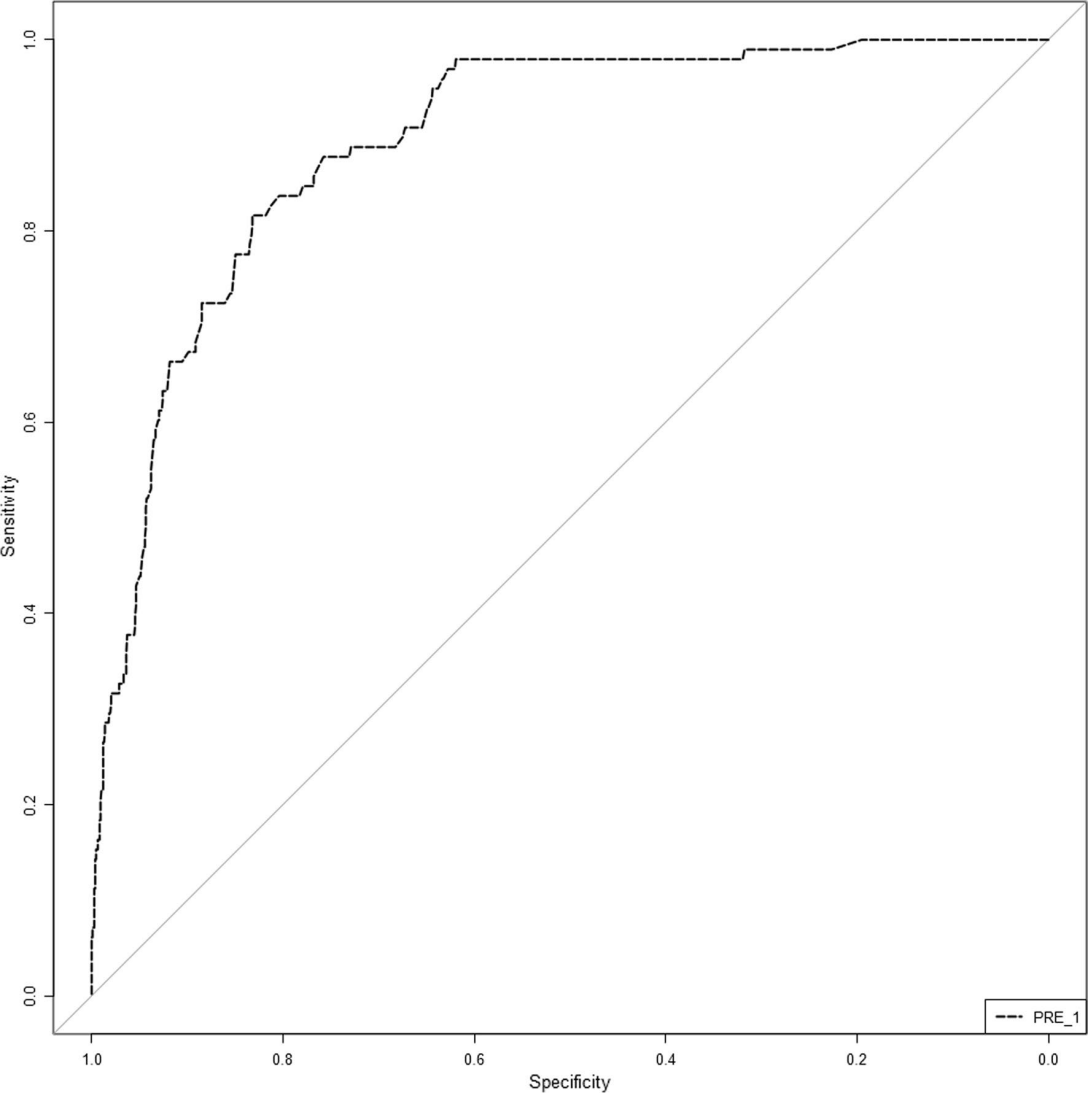
Receiver operating characteristic curve for the prediction model. Area under the curve was 0.894 (95% confidence interval 0.865–0.923)

**Fig. 3:**
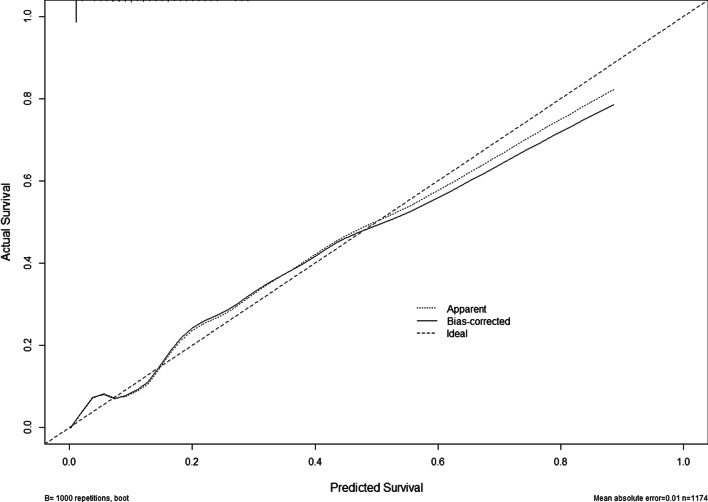
Calibration of the nomogram for early postoperative bowel obstruction. The x-axis shows the predicted probability of early postoperative bowel obstruction, and the y-axis shows the observed probability of early postoperative bowel obstruction

Based on previous results, a nomogram that included five before-mentioned risk factors was constructed to predict postoperative bowel obstruction (Fig. 1). Each risk factor was listed separately with corresponding number points assigned to a given magnitude of the risk factors. All risk factors were projected upward to obtain the matching points on the small ruler and the points assigned to the corresponding predictors were added up to obtain the total points. Then, the corresponding risk of early postoperative bowel obstruction was obtained by drawing down a vertical line from the “total points” axis to the “risk” axis to calculate. The value of each of these variables was given a score on the point scale axis. For example, one patient diagnosed hypothyroidism (matching 17.5 points) and COPD (matching 10 points) before this surgery, the duration of antibiotics was 10 days matching 10 points, the time to postoperative feeding was 4 days matching 10 points and had the probiotic indications matching 40 points. The total points were 87.5 points and the corresponding risk of early postoperative bowel obstruction was about 70%. The area under the curve (AUC) was 0.894 (95% confidence interval 0.865–0.923) (Fig. 2) and the calibration curve was presented in Fig. 3.

## Discussion

Postoperative bowel obstruction was a quite common complication after colorectal surgery [10]. Previous studies showed that risk factors of prolonged or early postoperative bowel obstruction for patients after colorectal surgery [11–13]. But few studies analyzed multiple risk factors for early postoperative bowel obstruction and no one previous study was found focusing on the prediction model of early postoperative bowel obstruction [14]. As far as we knew, our study was the very first to establish a nomogram to predict patient-specific factors for early postoperative bowel obstruction with the high discriminative ability and excellent calibration. In this study, we identified COPD, hypothyroidism, probiotic indications, duration of antibiotics, and time of first postoperative feeding as potential risk factors and created a statistical predictive model for early postoperative bowel obstruction.

Recent findings highlighted that gut microbiota might play an important role in shaping lung inflammation [15]. Both imbalance of gut microflora, which led to the release of intestinal endotoxin, and translocation of intestinal flora, which led to the release of inflammatory mediators could directly or indirectly promote the beginning and development of COPD [16]. Several studies found that COPD was the only preoperative morbidity predictive of complications after colorectal cancer resection [17, 18]. A history of COPD was an independent risk factor of postoperative 30-day morbidity, mortality, and hospital duration of stay in patients who underwent colectomy, small bowel resection, and appendectomy [19]. In that study, COPD was also found as an independent predictor for early postoperative bowel obstruction in patients who underwent colorectal surgery [20].

Patients with chronic constipation and abdominal distension should be considered with the diagnosis of hypothyroidism in 1969 [21]. Goto et al. [22] compared the daily stool volume of rats between the hypothyroidism group and sham-operated group. They found [22] that hypothyroidism impaired colonic motility and function as frequently seen in rats. Another case report [23] reported that a patient got intestinal symptoms from hypothyroidism in which previous conventional examinations were negative. In our study, the incidence of early postoperative bowel obstruction was higher in patients with hypothyroidism. Hypothyroidism might impaired gut motility [24].

During recent years the impact of antibiotic agents on the intestinal microflora has been investigated in patients undergoing colorectal surgery [25]. Prophylactic antibiotics are effective in preventing surgical wound infections [26]. Antibiotics impact the intestinal microflora depends on the agents’ spectrum and concentration [27]. Antibiotics usually had significant additional effects on many tissues. The study comparing the contractile responses of ileum smooth muscle to different agonists in adult guinea pigs indicated that antibiotics may impair small bowel smooth muscle contractility, which was the implication for the long-term use of parenteral antibiotics in the postoperative period [28]. In our study, the duration of antibiotics (11.92 ± 9.10 days) was longer in the early postoperative bowel obstruction group (OR 1.10, 95%CI 1.05–1.15). According to the guideline of probiotics use [29], the three most common clinical indications were prevention of antibiotic-related side effects, treatment of antibiotic-related side effects, and irritable bowel syndrome. Our study indicated that patients with antibiotic-related side effects such as diarrhea and abdominal distension (also as probiotic indications), were potential victims of early postoperative bowel obstruction. But there was no difference between patients with and without using probiotics.

A meta-analysis [30] evaluating the effect on the early time of oral feeding after upper gastrointestinal surgery found that early postoperative oral feeding reduced the length of hospital stay and decreased relevant complications as anastomotic leak and incidence of nasogastric tube reinsertion. Toledano et al. [31] showed that early oral feeding reduced the time to first flatus and first ostomy output compared with traditional diet in the patients who had an ostomy creation, which may promote the recovery of bowel function. In our study, time to first postoperative feeding was an independent risk factor of early postoperative bowel obstruction.

In previous studies, mechanical bowel preparation was routinely used to improve postoperative recovery of colon surgery [32]. But bowel cleaning with oral antibiotics can reduce the incidence of surgical site infection, anastomotic leakage, and other morbidity compared with mechanical bowel preparation [33–37] for patients undergoing colorectal surgery. Recently, Koskenvuo et al. recruited a multicenter, parallel, single-blind trial to fund that oral antibiotics bowel preparation cannot reduce the occurrence of surgical site infection or overall morbidity after colonic surgery [38]. There was no significant difference in mechanical bowel preparation with or without oral antibiotics for early postoperative bowel obstruction.

Plasma albumin is involved in the transport of human metabolites, maintenance of colloid osmotic pressure, and other physiological functions [39]. Plasma albumin value is performed to evaluate the postoperative nutritional status [40]. Another study showed no significant difference in the postoperative day of flatus or postoperative day of intake between patients with albumin levels dropped below 3.5 g/dl and patients with albumin greater or equal to 3.5 g/dL [41]. Our study showed that hypoproteinemia may not be an independent risk factor of early postoperative bowel obstruction for patients after colorectal surgery.

Analgesia is becoming more acceptable by surgical patients. Opioids are the most commonly used for analgesia. Almost 94.6% of patients choose opioids for analgesia in our study. But opioids have numerous side effects such as breath inhibition, nausea, vomiting, ileus, and urinary retention [42]. One study showed that intravenous acetaminophen helps to reduce opioids consumption, which reduced the time to return bowel function for patients undergoing colorectal surgery [43]. In our study, both analgesic protocol and duration of analgesia were not the risk factors of early postoperative bowel obstruction for patients who underwent colorectal surgery.

It is that the incidence of postoperative morbidity mortality increased in elderly patients [44], which elderly patients were more likely to present with later-stage disease. ASA classification is associated with postoperative morbidity in patients undergoing major abdominal cancer surgery [45]. ASA score predicts postoperative length of stay and complication risk [46]. Age and ASA classification may increase the incidence of early postoperative bowel obstruction after benefactor analysis but was not an independent factor for early postoperative bowel function after logistic regression analysis in this study.

We investigated multiple factors that may be associated with early postoperative bowel obstruction. But there were also some limitations because the data were collected retrospectively: firstly, the data varied widely because there are different wards in the department of gastrointestinal surgery. More and more surgeons are following the principle of Enhanced Recovery after Surgery (ERAS) protocol for perioperative management. However, there are also someone choose traditional methods for perioperative management in our department. Secondly, the history of constipation that reflect the status of bowel function was usually neglected in the real clinical practice. Finally, it was still unclear whether the predictive model could be applied in all patients because our data were collected from China. Nevertheless, we think our nomogram could help the postoperative management of patients undergoing colorectal surgery.

## Conclusion

To our knowledge, this study presented that COPD, hypothyroidism, probiotic indications, duration of antibiotics, and time of first postoperative feeding were the independent risk factors for early postoperative bowel obstruction after colorectal surgery. A nomogram based on the above key factors was established to predict the risk of early postoperative bowel obstruction, which might help to improve postoperative management in patients.

## Data Availability

The datasets are not publicly available due to privacy or ethical restrictions but are available from the corresponding author on reasonable request.

## Abbreviations

BMI: Underlying disease
CHD: Coronary heart disease
COPD: Chronic obstructive pulmonary disease
ASA: American Society of Anesthesiologists
CT: Computer tomography
OR: Odds ratios
CI: Confidence intervals
AUC: Area under the curve

## References

[1] Muffly TM, Ridgeway B, Abbott S, Chmielewski L, Falcone T (2012). Small bowel obstruction after hysterectomy to treat benign disease. J Minim Invasive Gynecol.

[2] Fevang BT, Fevang J, Stangeland L, Soreide O, Svanes K, Viste A (2000). Complications and death after surgical treatment of small bowel obstruction: a 35-year institutional experience. Ann Surg.

[3] Miller G, Boman J, Shrier I, Gordon PH (2000). Etiology of small bowel obstruction. Am J Surg.

[4] Edna TH, Bjerkeset T (1998). Small bowel obstruction in patients previously operated on for colorectal cancer. Eur J Surg = Acta Chir.

[5] Pickleman J, Lee RM (1989). The management of patients with suspected early postoperative small bowel obstruction. Ann Surg.

[6] Antosh DD, Grimes CL, Smith AL, Friedman S, McFadden BL, Crisp CC, Allen AM, Gutman RE, Rogers RG (2013). A case-control study of risk factors for ileus and bowel obstruction following benign gynecologic surgery. Int J Gynaecol Obstet: Off Organ Int Fed Gynaecol Obstet.

[7] Chapman SJ, Thorpe G, Vallance AE, Harji DP, Lee MJ, Fearnhead NS (2019). Systematic review of definitions and outcome measures for return of bowel function after gastrointestinal surgery. BJS Open.

[8] Gómez-Izquierdo JC, Feldman LS, Carli F, Baldini G (2015). Meta-analysis of the effect of goal-directed therapy on bowel function after abdominal surgery. Br J Surg.

[9] Wilson MS, Hawkswell J, McCloy RF (1998). Natural history of adhesional small bowel obstruction: counting the cost. Br J Surg.

[10] Matter I, Khalemsky L, Abrahamson J, Nash E, Sabo E, Eldar S (1997). Does the index operation influence the course and outcome of adhesive intestinal obstruction?. Eur J Surg = Acta Chir.

[11] Quiroga-Centeno AC, Jerez-Torra KA, Martin-Mojica PA, Castañeda-Alfonso SA, Castillo-Sánchez ME, Calvo-Corredor OF, Gómez-Ochoa SA (2020). Risk factors for prolonged postoperative ileus in colorectal surgery: a systematic review and meta-analysis. World J Surg.

[12] Nakajima J, Sasaki A, Otsuka K, Obuchi T, Nishizuka S, Wakabayashi G (2010). Risk factors for early postoperative small bowel obstruction after colectomy for colorectal cancer. World J Surg.

[13] Wang XJ, Chi P, Lin HM, Lu XR, Huang Y, Xu ZB, Huang SH, Sun YW, Ye DX (2018). Risk factors for early postoperative small bowel obstruction after elective colon cancer surgery: an observational study of 1,244 consecutive patients. Dig Surg.

[14] Masoomi H, Kang CY, Chaudhry O, Pigazzi A, Mills S, Carmichael JC, Stamos MJ (2012). Predictive factors of early bowel obstruction in colon and rectal surgery: data from the Nationwide Inpatient Sample, 2006–2008. J Am Coll Surg.

[15] McAleer JP, Kolls JK (2018). Contributions of the intestinal microbiome in lung immunity. Eur J Immunol.

[16] Kim HJ, Li H, Collins JJ, Ingber DE (2016). Contributions of microbiome and mechanical deformation to intestinal bacterial overgrowth and inflammation in a human gut-on-a-chip. Proc Natl Acad Sci USA.

[17] Leung AM, Gibbons RL, Vu HN (2009). Predictors of length of stay following colorectal resection for neoplasms in 183 veterans affairs patients. World J Surg.

[18] Lemmens VE, Janssen-Heijnen ML, Houterman S, Verheij KD, Martijn H, van de Poll-Franse L, Coebergh JW (2007). Which comorbid conditions predict complications after surgery for colorectal cancer?. World J Surg.

[19] Fields AC, Divino CM (2016). Surgical outcomes in patients with chronic obstructive pulmonary disease undergoing abdominal operations: an analysis of 331,425 patients. Surgery.

[20] Millan M, Biondo S, Fraccalvieri D, Frago R, Golda T, Kreisler E (2012). Risk factors for prolonged postoperative ileus after colorectal cancer surgery. World J Surg.

[21] Cole P, Petrie JC, Bewsher PD (1969). Intestinal obstruction and hypothyroidism. Br Med J.

[22] Goto S, Billmire DF, Grosfeld JL (1992). Hypothyroidism impairs colonic motility and function. An experimental study in the rat. Eur J Pediatric Surg.

[23] Bassotti G, Pagliacci MC, Nicoletti I, Pelli MA, Morelli A (1992). Intestinal pseudoobstruction secondary to hypothyroidism. Importance of small bowel manometry. J Clin Gastroenterol.

[24] Hays MT (1988). Thyroid hormone and the gut. Endocr Res.

[25] Brismar B, Edlund C, Malmborg AS, Nord CE (1990). Ecological impact of antimicrobial prophylaxis on intestinal microflora in patients undergoing colorectal surgery. Scand J Infect Dis Suppl.

[26] Classen DC, Evans RS, Pestotnik SL, Horn SD, Menlove RL, Burke JP (1992). The timing of prophylactic administration of antibiotics and the risk of surgical-wound infection. N Engl J Med.

[27] Nord CE (1990). Studies on the ecological impact of antibiotics. Eur J Clin Microbiol Infect Dis.

[28] Ceran C, Karadas B, Kaya T, Arpacik M, Bagcivan I, Sarac B (2006). Do antibiotics contribute to postoperative ileus? Contractile responses of ileum smooth muscle in Guinea pigs to long-term parenteral ceftriaxone and ampicillin. ANZ J Surg.

[29] Draper K, Ley C, Parsonnet J (2017). Probiotic guidelines and physician practice: a cross-sectional survey and overview of the literature. Benef Microbes.

[30] Willcutts KF, Chung MC, Erenberg CL, Finn KL, Schirmer BD, Byham-Gray LD (2016). Early oral feeding as compared with traditional timing of oral feeding after upper gastrointestinal surgery: a systematic review and meta-analysis. Ann Surg.

[31] Toledano S, Sackey J, Willcutts K, Parrott JS, Tomesko J, Al-Mazrou AM, Kiran RP, Brody RA (2019). Exploring the differences between early and traditional diet advancement in postoperative feeding outcomes in patients with an ileostomy or colostomy. Nutr Clin Pract: Off Publ Am Soc Parent Enteral Nutr.

[32] Wind J, Polle SW, Jin PHPFK, Dejong CHC, Von Meyenfeldt MF, Ubbink DT, Gouma DJ, Bemelman WA (2006). Systematic review of enhanced recovery programmes in colonic surgery. Acute Pain.

[33] Condon RE, Bartlett JG, Greenlee H, Schulte WJ, Ochi S, Abbe R, Caruana JA, Gordon HE, Horsley JS, Irvin G (1983). Efficacy of oral and systemic antibiotic prophylaxis in colorectal operations. Arch Surg.

[34] Takesue Y, Yokoyama T, Akagi S, Ohge H, Murakami Y, Sakashita Y, Miyamoto K, Uemura K, Itaha H, Matsuura Y (2000). A brief course of colon preparation with oral antibiotics. Surg Today.

[35] Rollins K, Javanmard-Emamghissi H, Acheson A, Lobo D (2018). The role of oral antibiotic preparation in elective colorectal surgery: a meta-analysis. Ann Surg.

[36] Fry DE (2011). Colon preparation and surgical site infection. Am J Surg.

[37] Espin Basany E, Solís-Peña A, Pellino G, Kreisler E, Fraccalvieri D, Muinelo-Lorenzo M, Maseda-Díaz O, García-González JM, Santamaría-Olabarrieta M, Codina-Cazador A (2020). Preoperative oral antibiotics and surgical-site infections in colon surgery (ORALEV): a multicentre, single-blind, pragmatic, randomised controlled trial. Lancet Gastroenterol Hepatol.

[38] Koskenvuo L, Lehtonen T, Koskensalo S, Rasilainen S, Klintrup K, Ehrlich A, Pinta T, Scheinin T, Sallinen V (2019). Mechanical and oral antibiotic bowel preparation versus no bowel preparation for elective colectomy (MOBILE): a multicentre, randomised, parallel, single-blinded trial. The Lancet.

[39] Adams HA, Piepenbrock S, Hempelmann G (1998). Volume replacement solutions–pharmacology and clinical use. Anasthesiol Intensivmed Notfallmed Schmerzther: AINS.

[40] Xiao-Bo Y, Qiang L, Xiong Q, Zheng R, Jian Z, Jian-Hua Z, Qian-Jun Z (2014). Efficacy of early postoperative enteral nutrition in supporting patients after esophagectomy. Minerva Chir.

[41] Woods MS, Kelley H (1993). Oncotic pressure, albumin and ileus: the effect of albumin replacement on postoperative ileus. Am Surg.

[42] Kehlet H, Dahl JB (1993). The value of "multimodal" or "balanced analgesia" in postoperative pain treatment. Anesth Analg.

[43] Aryaie AH, Lalezari S, Sergent WK, Puckett Y, Juergens C, Ratermann C, Ogg C (2018). Decreased opioid consumption and enhance recovery with the addition of IV Acetaminophen in colorectal patients: a prospective, multi-institutional, randomized, double-blinded, placebo-controlled study (DOCIVA study). Surg Endosc.

[44] Colorectal Cancer Collaborative Group (2000). Surgery for colorectal cancer in elderly patients: a systematic review. Lancet.

[45] Hightower CE, Riedel BJ, Feig BW, Morris GS, Ensor JE, Woodruff VD, Daley-Norman MD, Sun XG (2010). A pilot study evaluating predictors of postoperative outcomes after major abdominal surgery: physiological capacity compared with the ASA physical status classification system. Br J Anaesth.

[46] Jacqueline R, Malviya S, Burke C, Reynolds P (2006). An assessment of interrater reliability of the ASA physical status classification in pediatric surgical patients. Paediatr Anaesth.

